# A Metacognitive Perspective of Visual Working Memory With Rich Complex Objects

**DOI:** 10.3389/fpsyg.2020.00179

**Published:** 2020-02-25

**Authors:** Tomer Sahar, Yael Sidi, Tal Makovski

**Affiliations:** ^1^Department of Psychology and Education, The Open University of Israel, Ra’anana, Israel; ^2^Department of Psychology, University of Haifa, Haifa, Israel

**Keywords:** subjective judgment, real-world objects, confidence, meaning, appearance errors

## Abstract

Visual working memory (VWM) has been extensively studied in the context of memory capacity. However, less research has been devoted to the metacognitive processes involved in VWM. Most metacognitive studies of VWM studies tested simple, impoverished stimuli, whereas outside of the laboratory setting, we typically interact with meaningful, complex objects. Thus, the present study aimed to explore the extent to which people are able to monitor VWM of real-world objects that are more ecologically valid and further afford less inter-trial interference. Specifically, in three experiments, participants viewed a set of either four or six memory items, consisting of images of unique real-world objects that were not repeated throughout the experiment. Following the memory array, participants were asked to indicate where the probe item appeared (Experiment 1) whether it appeared at all (Experiment 2) or whether it appeared and what was its temporal order (Experiment 3). VWM monitoring was assessed by subjective confidence judgments regarding participants’ objective performance. Similar to common metacognitive findings in other domains, we found that subjective judgments overestimated performance and underestimated errors, even for real-world, complex items held in VWM. These biases seem not to be task-specific as they were found in temporal, spatial, and identity VWM tasks. Yet, the results further showed that meaningful, real-world objects were better remembered than distorted items, and this memory advantage also translated to metacognitive measures.

## Introduction

To what degree does one have access to own mental processes? The body of research termed *Metacognition* aims to answer this question. The field of metacognition refers to “thinking about thinking” (Flavell, 1979) and it deals with the evaluation and monitoring of cognitive processes and the control and regulation of these processes (see Koriat, 2007 for review). Broadly speaking, monitoring of cognitive processes refers to one’s awareness of the operation of a specific cognitive process while it occurs. From an experimental perspective, monitoring is usually assessed by collecting participants’ direct (subjective) confidence judgments regarding the relevant process, and matching that with the actual outcome. Consequently, metacognitive studies often find that monitoring may be based on heuristics: a pragmatic but not necessarily optimal approach to generate subjective judgments, as in certain situations, they may prove unreliable and lead to biased decisions (Tversky and Kahneman, 1974).

To assess the degree to which monitoring coincides with the actual performance, two central measures are used: calibration and resolution (Fleming and Lau, 2014; Fiedler et al., 2019). Calibration, or absolute accuracy, refers to the gap between subjective confidence judgments and task performance scores (e.g., correct responses). Thus, calibration is maximized when the proportion of correct responses equals to the subjective confidence judgments given by the observer, and the absolute difference is zero. That is, subjective confidence ratings equal to the actual performance. An overconfidence bias occurs when subjective confidence exceeds task scores—as the observer overestimates her performance. Conversely, an underconfidence bias occurs when high performance is underestimated.

Resolution, or relative accuracy, is the extent to which confidence judgments vary between a correct or incorrect response. This is measured as a correlation between confidence and accuracy. Resolution is maximized when high performance is predicted by high confidence judgments and low performance is predicted by low confidence judgments (Ackerman and Goldsmith, 2011, for reviews, see Schwartz and Efklides, 2012; Goldsmith et al., 2014). Note that calibration and resolution are two independent measures. Calibration reflects the extent of deviation from being subjectively accurate in confidence judgments, whereas resolution is a correlation that reflects the extent of how judgments represent and change with performance.

The current study aimed at examining visual working memory (VWM) from a metacognitive perspective. VWM is considered to be a fundamental, capacity-limited on-line buffer, and individual differences in this ability are related to high cognitive functions, such as intelligence (Luck and Vogel, 2013). Hence, understanding how people access and assess the content held in VWM can shed new light on the mechanisms underlying VWM processes. Furthermore, the relationship between working memory and metacognitive abilities is likely to be bi-directional. For example, Komori (2016) showed that in a dual-task setting, observers with high working memory capacity made more accurate judgments about their performance than observers with low capacity. On the flip side, researchers are also relying on the assumption that observers have accurate metacognitive reports and use that to assess VWM processes (Adam et al., 2017). Thus, studying metacognitive processes within VWM can gain valuable insights into both VWM and metacognitive processes.

Metacognitive studies of VWM have mainly examined the accuracy of subjective estimations of VWM limit and the extent that subjective and objective visual knowledge dissociate from one another. The correspondence of objective VWM measures and subjective judgments showed that, overall, subjective judgments reliably reflect (at least to some extent) VWM content and objective visual information (Rademaker et al., 2012; Vandenbroucke et al., 2014; Samaha and Postle, 2017; Suchow et al., 2017). Yet, other studies have stressed the separability of objective visual information and subjective judgments (Bona et al., 2013; Bona and Silvanto, 2014; Vlassova et al., 2014; Maniscalco and Lau, 2015). For instance, Adam and Vogel (2017) showed that while subjective judgments predicted some variation in memory performance, observers were consistently unaware of their own memory failures.

One issue of measuring metacognitive processes in VWM is the repeated use of a limited set of simple stimuli (e.g., colors, orientations) in VWM tasks. This results in a narrow, homogeneous stimuli space and increases the likelihood of proactive interference. The outcome of proactive interference is that items from previous trials are harder to reject, and are mistakenly reported as if they appeared in the current trial (e.g., Keppel and Underwood, 1962; Hartshorne, 2008; Makovski and Jiang, 2008; Makovski, 2016; but see Lin and Luck, 2012). Thus, without accounting for these errors, studies might inaccurately estimate VWM performance, and more importantly for the current purposes, they might impair our ability to adequately assess the metacognitive processes involved in VWM because both subjective and objective performance are likely to be contaminated by information encountered in previous trials.

One way to minimize proactive interference is by using real-world objects instead of simple stimuli. These stimuli afford to test numerous distinct items without repetition throughout the experiment (Endress and Potter, 2014; Makovski, 2016; Shoval et al., 2019). Testing real-world objects in VWM tasks further bears an ecological benefit as we typically interact with meaningful, rich, complex objects and not with impoverished stimuli such as color patches. Accordingly, recent findings showed that the visual and semantic heterogeneity of meaningful objects leads to an improved VWM performance and extend the typical limit of VWM capacity (Brady et al., 2016; Shoval et al., 2019). However, it is still unknown how accurate people are in monitoring VWM of rich, real-world objects.

The goal of the current study was to explore observers’ ability to monitor VWM processes using distinct complex stimuli and various VWM tasks. Three experiments were conducted in order to reveal the correspondence between objective and subjective memory performance while minimizing proactive interference by using non-repeating images of real-world objects. Specifically, we measured observers’ resolution and calibration while they were performing VWM tasks with unique (i.e., presented only once in the task) and distinct real-world objects. This allowed us to estimate the metacognitive abilities of VWM across three domains (e.g., spatial, identity, temporal) with minimal interference from the information shown in previous trials.

## Experiment 1

The aim of the first experiment was to examine spatial VWM performance from a metacognitive perspective. Thus, on each trial, participants memorized a set of six images of real-world objects, presented sequentially at distinct locations (Makovski, 2016). After a short retention period, one of the presented images appeared and participants were asked to indicate the item’s location. Next, they were asked to evaluate their confidence by indicating the degree of certainty that they chose the correct item’s location on a 0–100 scale. This allowed us to assess both subjective and objective performance and thereby estimate resolution and calibration.

### Method

#### Participants

Participants were students (age: 18–35) from the Open University of Israel who took part in the experiment for course credit. All had normal or corrected-to-normal visual acuity and were without learning disabilities or attention disorders. Power calculation showed that a minimum sample size of 20 participants provided a power of 0.8 for detecting a Cohen’s d effect size of 0.66 using a two-tailed paired samples *t*-test. Twenty-two participants completed Experiment 1 (19 females, mean age = 27).

#### Materials and Stimuli

**FIGURE 1:**
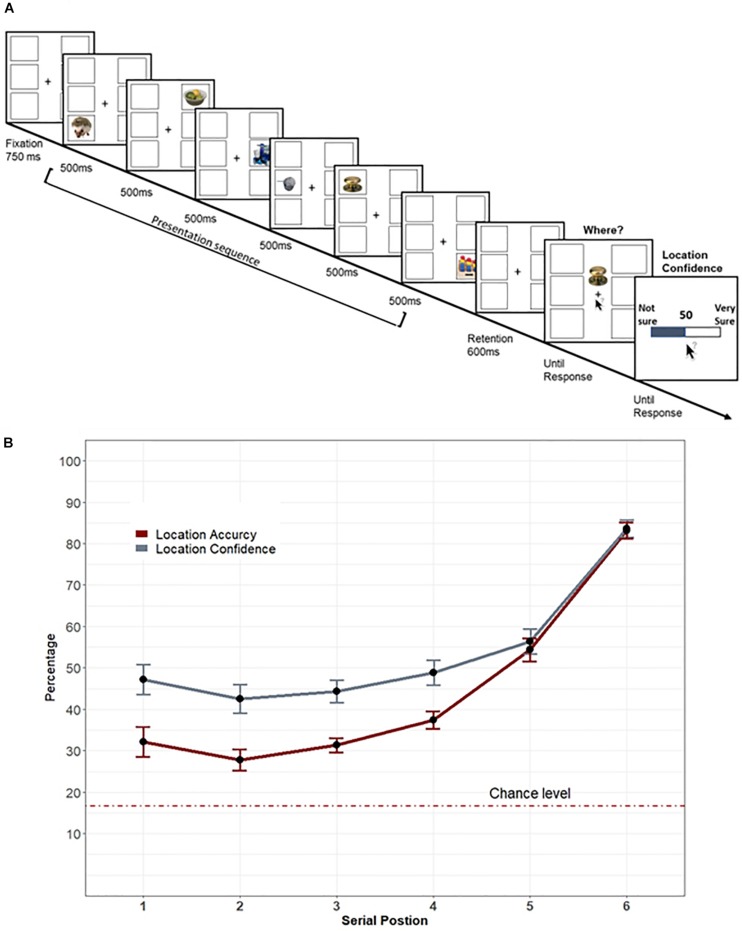
**(A)** Illustration of Experiment 1’s trial’s sequence: Each trial began with blank place-holders. The presentation sequence consisted of six items, each at a distinct location. After the final stimulus and between the presentation of the probe, a blank place-holders display was shown for 600 ms. Following this short retention, participants were asked to indicate where the probed item appeared and to rate their confidence regarding their response. **(B)** Experiment 1’s results: Mean confidence (gray line) and the mean percentage of correct location (red line) plotted as a function of the probed-item’s serial position during the presentation sequence. Error bars represent standard error of the mean.

The task was created and implemented with MATLAB software (MathWorks Inc., Natick, MA, United States, 2010) and Psychtoolbox (Brainard, 1997) on a 23.5” Eizo Foris monitor (1920 × 1080, 120 Hz) and a standard PC. Participants were tested individually in a dim room. They sat approximately 50 cm from the screen. A black fixation cross (0.96°) was presented at the center of a white background screen. Two columns of three black-frame empty squares (5.6° × 5.6°) served as place-holders (located 14° to the left and right of fixation, and 14° above, at fixation level, and 14° below the fixation, Figure 1). The image set included 1200 images of real-world objects (4.8° × 4.8°) drawn from a previously published set (Brady et al., 2008^1^). Confidence judgments were collected by scrolling with the mouse over a rectangle bar (40° × 1.9°). The initial position of the cursor was at the middle of the bar (i.e., at 50%). The bar was interactively filled with the color blue from its left edge to the position of the cursor. The percentage of the filled area, from 0 to 100, served as a numeric indicator for confidence and it was presented above the rectangle. Participants finalized their judgment response by pressing the space key. Note that responding without moving the cursor was impossible, and a response of 50% was not allowed.

#### Procedure

The trial began with a 950 ms fixation and place-holders display that remained visible throughout the trial. Each trial consisted of six unique images, randomly drawn in each trial for each subject. Each image appeared in isolation within a distinct place-holder for 500 ms. The items appeared sequentially in random order and after the last image was shown, a fixation cross was displayed for 600 ms. Then, the probe item, which was always one of the six items presented in that trial, appeared above fixation together with the six empty place-holders and the mouse cursor at fixation. The probe item was evenly and randomly chosen from the six possible locations and six serial positions. Participants were instructed to indicate the place-holder in which the probe item appeared by clicking on its position using the mouse. There was no time limit for this task and only accuracy was emphasized. After a response was registered, participants were instructed to indicate their subjective confidence that they made a correct response by scrolling with the mouse over a 0 (“*not-sure*”) to 100 (“*very sure*”) scale. A numeric value of confidence was accordingly shown, and the participants were instructed to choose any value that reflected their subjective confidence except for 50%. The next trial began after 500 ms of a blank display (Figure 1A).

Participants performed 180 experimental trials (five trials in each of the six locations, six serial-positions combinations), preceded by eight practice trials. Every 36 trials participants could take a short break.

### Results

#### Accuracy

Figure 1B depicts performance as a function of serial position. The overall accuracy reflected moderately poor performance, but was above chance level [16.6%, *M* = 44.3%, *SD* = 22.9, *t*(21) = 18.2, *p* < 0.001]. A repeated-measures analysis of variance (ANOVA, Greenhouse–Geisser corrected) of accuracy as a function of the probed-item serial position was significant, *F*(2.84,59.7) = 82.063, *p* < 0.001, η_p_^2^ = 0.796. Bonferroni corrected comparisons showed that accuracy was best for the last item to (the 6th item, all *p*’s < 0.001). The second to last item (the 5th item) was also better than all previous positions (all *p*’s < 0.001). There was no other significant difference between positions 1–4 (all *p*’s > 0.1) except that the fourth item was better than the second item (*p* = 0.017). These results reflect a typical recency effect as the locations of the last two items were better remembered than the location of the first four items (Broadbent and Broadbent, 1981).

#### Confidence

Similar to accuracy, a repeated-measures ANOVA of confidence ratings (Figure 1B) as a function of the probed-item serial position revealed a significant effect, *F*(2.79,58.75) = 77.872, *p* < 0.001, η_p_^2^ = 0.788. Bonferroni corrected *post hoc* comparisons showed that confidence was largest for the last presented item (all *p*’s < 0.001). The confidence of the second to last presented item was also larger than all previously presented items (all *p*’s < 0.046). No other significant difference was found (all *p*’s > 0.08).

#### Calibration

Calibration was calculated as the difference between confidence and accuracy in each serial position of each subject. Repeated-measures ANOVA of calibration as a function of serial position revealed a significant main effect, *F*(5,105) = 9.063, *p* < 0.001, η_p_^2^ = 0.301. To further examine the source of the overconfidence bias, *post hoc* Bonferroni corrected comparisons showed that the last item significantly differed from the third, second, and first items (all *p*’s < 0.033). The fifth item differed from all previous items (all *p*’s < 0.028). No other comparisons were significant. Bayesian one-sample *t*-test further showed a reliable and positive difference from zero for the first four items (BF = 36, 13, 21, 5.4, respectively), but did not show a reliable difference from zero for the last two presented items (BF = 0.26, 0.22, respectively). This suggests that the overconfidence bias was driven from the first four items, whereas observers were well-calibrated for the last two items (see Figure 1B).

#### Resolution

For each participant, a resolution was calculated as the Gamma correlation coefficient (i.e., Goodman–Kruskal correlation) between accuracy and confidence (Nelson, 1984) collapsed across all serial positions. The averaged resolution across participants was moderate (*M* = 0.521, *SD* = 0.1), suggesting that observers’ discrimination between the better- and less-remembered location of the probed item was only accurate to some extent.

### Discussion

The results of Experiment 1 showed that participants’ sensitivity to their performance in the VWM task was moderate—as reflected by their resolution. However, this estimation (0.521) seems to be numerically larger than correlations previously reported in other metacognitive studies of VWM, which varied between 0.19 and 0.47 (0.22–0.39, Thomas et al., 2012; 0.19–0.43, Yue et al., 2013; 0.47, Adam and Vogel, 2017; but see Masson and Rotello, 2009). We also found that the calibration was highly influenced by the item’s serial position as observers were overconfident in the first four items but well-calibrated in the last two items.

In the current experiment, we asked observers about the item’s location and not about the memory of the item itself. That is, the objective and subjective measures were only based on the spatial memory of the item (where the item was presented). However, a crucial aspect of memory is the explicit access to the item’s identity, which is also often used as a measure of memory performance (e.g., “was this chair presented?”). Therefore, in Experiment 2, we turn to directly examine whether participants explicitly remember the probed item and particularly their confidence that the probed item appeared.

## Experiment 2

While we usually interact with both the item’s identity and its location, they are not necessarily recalled together nor do they decay together in an obligatory manner (Köhler et al., 2001; Pertzov et al., 2012). Thus, testing spatial memory alone, as in the previous experiment, does not provide a full view of the metacognitive abilities of VWM. Specifically, it remains unclear whether people can accurately assess their VWM when it is based on the item’s identity.

Several changes were therefore done in Experiment 2. First, the presentation set-size was reduced to four items to ensure that the capacity limit was not exceeded. As in Experiment 1, each item appeared at a distinct location, and items were not repeated throughout the experiment. After the presentation sequence, observers were asked to indicate whether they explicitly remember that the probed item appeared and to rate their confidence regarding the item’s appearance. Importantly, the probed item was always an item from the presentation sequence. Afterward, they were asked to indicate its location. When participants reported that the probed item did not appear, an “appearance error” was registered but the trial continued the same. That is, participants were asked to guess a possible location and were not told anything about whether the item actually appeared or not (note that the item always appeared). This allowed us examine the location accuracy in those trials where participants reported that they do not remember that the item appeared (i.e., its identity). Note that in this experiment, we focused on participants’ reports and confidence ratings that the item appeared and thus we did not measure the confidence in knowing where the item appeared (as was in Experiment 1).

### Method

#### Participants

Thirty-six new participants from the Open University of Israel completed Experiment 2 (25 females, mean age 25.3).

#### Materials, Stimuli, and Procedure

**FIGURE 2:**
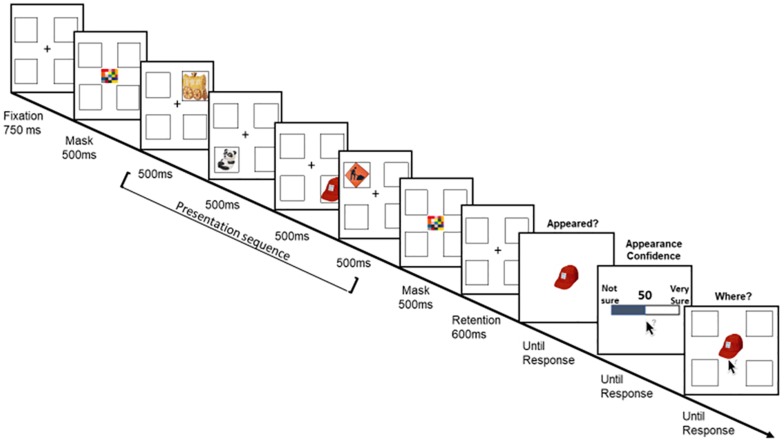
A schematic illustration of Experiment’s 2 trial’s sequence.

Unique non-repeated images were drawn from the same set used in Experiment 1. Because memory set-size was reduced, and to avoid verbal coding, articulatory suppression was included in the task. Specifically, each trial began with a randomly drawn word (out of 24 Hebrew words, three letters, two syllables) that participants were asked to repeat aloud throughout the presentation sequence. Participants initiated the trial by pressing the mouse button when they were ready. Then, the fixation with the place-holders display was shown for 750 ms. The place-holders display consisted of four black-frame squares (2 by 2, 5.6 × 5.6 each) 12° to the left and right of fixation and 12° above and below fixation. To match the presentation condition to Experiment 1 (in terms of forward and backward masking), a multi-colored square (4.8 × 4.8) was presented for 500 ms at the center of the display before the first item and after at the fourth item. After 600 ms of a retention interval, the probed item appeared, and participants were asked to indicate whether or not they remember this item by pressing the keyboard keys “1” or “2,” respectively. Importantly, the probed item was always one item from the sequence. Note that when participants reported that the item did not appear, the trial continued the same. After this response, participants were asked to rate their subjective confidence that the probed item appeared or not using the same method in Experiment 1. After their appearance confidence rating was registered, the probed item re-appeared at the center of the display together with the empty place-holders and the participants were asked to use the mouse to indicate the probed-item location. If participants indicated that the probed item did not appear, they were asked to guess its possible location. No confidence question was asked for this response (Figure 2).

Participants performed eight practice trials before continuing to 192 experimental trials (12 trials in each of the four locations, four serial positions combinations). In order to control for items’ memorability, all participants viewed the same presentation sequences and probed items, in the same order. Every 32 trials participants could take a short break and the session lasted approximately 30 min. In all other respects, the method was identical to Experiment 1.

### Results

#### Statistical Analyses

Due to the subjective nature of “appearance” (i.e., remembering an item), different participants produced different proportions (if any) of “not appeared” errors (as on each trial the probe item was always presented), range = 0–42%, median = 9.9%, SD = 10.04. To avoid excluding participants on the basis of balancing sample-sizes, we used mixed-effects models to analyze the data of “not-appeared” trials. For each outcome variable (i.e., appearance confidence, appearance calibration, location accuracy), we used a simple linear mixed-effects model (LMM) with only one main effect (serial position: 1–4) as a fixed effect and participants as a random effect.

The effects in this model were tested using the *lme* function of the *nlme* package (version 3.1 – 137, Pinheiro et al., 2019). The *F*-values and *p*-values (approximation by the degrees of freedom) of the effects were calculated by implementing the *ANOVA* function from the *stats* package (version 3.5.2, R Core Team, 2019). *Post hoc* comparisons are reported with Tukey adjustments.

**FIGURE 3:**
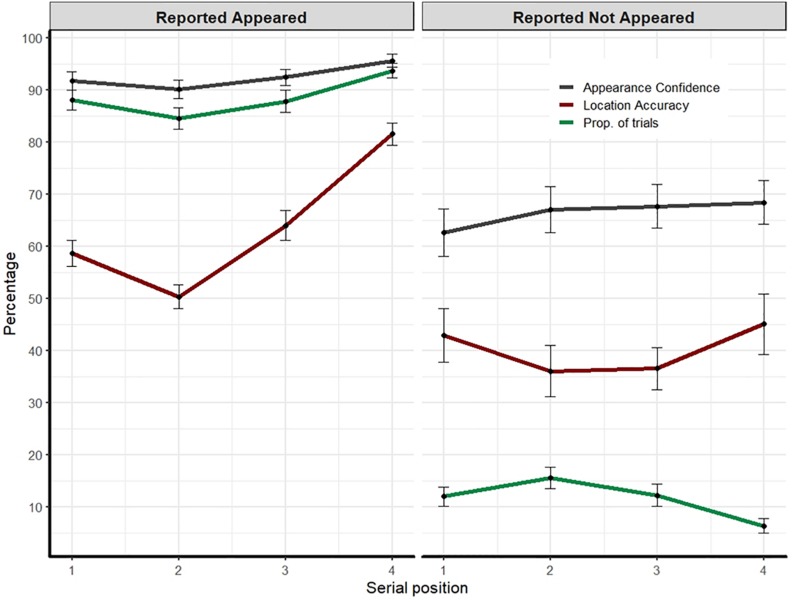
Experiment’s 2 results: Appearance confidence, mean correct location responses, and the proportion of trials as a function of participants’ appearance report (i.e., appear, did not appear) and the item’s serial position. Error bars represent one standard error of the mean.

For “appeared” trials (without the problem of uneven observations), we used, as before, repeated-measures ANOVAs with serial position as a within-subject factor and Greenhouse–Geisser corrected where needed. *Post hoc* comparisons were adjusted using Bonferroni correction. Figure 3 depicts the results.

#### Proportion of Trials and Appearance Errors

Overall, in about 89% of trials, participants reported correctly that they remember the probed item (Figure 3, left). The complement (M = 11%) reflects the proportion of appearance errors (Figure 3, right) which can be viewed as “misses” because the probed item was actually presented on each trial. These errors varied with serial position, *F*(3,105) = 14.26, *p* < 0.001, η_p_^2^ = 0.29. Bonferroni corrected *post hoc* comparisons showed that the last item was the most accurate (reported as appeared, all *p*’s < 0.008). The third item differed from the second item (*p* = 0.012). No other comparisons were significant. We now turn to describe the memory and metacognitive measures as a function of participants’ reports on the item’s appearance.

#### Reported Appeared

##### Appearance confidence

Appearance confidence was high (M = 92.4, SD = 9.1) and although numerically similar, differences in confidence as a function of the probed-item serial position were significant, *F*(1.9,69.4) = 18.9, *p* < 0.001, η_p_^2^ = 0.351. *Post hoc* comparisons showed that the confidence for the last item was the highest (all *p*’s = 0.001). The third item was higher than the second item (*p* = 0.004). The first item was also higher than the second (*p* = 0.041).

##### Appearance calibration

There was no significant effect of the item’s serial position on calibration *F*(3,105) = 2.005, *p* = 0.118. Overall, one-sample two-sided Bayesian *t*-test showed the average calibration was numerically close to zero but was inconclusively different than zero [M = 3.9, SD = 13.7, two-sided one-sample *t*-test, *t*(35) = 1.7, *p* = 0.095, BF = 0.675]. Nevertheless, the lack of overall overconfidence bias in these data should be taken with caution because of a ceiling effect as the performance was quite high.

##### Location accuracy

Overall, the averaged location memory was moderate (M = 63.6%, SD = 10.5) and a similar pattern of serial position emerged *F*(2.4,85.5) = 46.8, *p* < 0.001, η_p_^2^ = 0.573. The last item was the most accurate (all *p*’s < 0.001). The third item was better than the second item (*p* < 0.001). The first item was better than the second (*p* = 0.002).

#### Reported Not Appeared

We now turn to examine the appearance confidence and calibration, as well as the location accuracy, separately for the trials on which participants erroneously reported that the probed item did not appear.

##### Appearance confidence

The overall confidence that the probed item did not appear was relatively high (M = 66.2, SD = 26.01) and did not differ across serial positions, *F*(1,87) < 1, *p* > 0.1.

##### Appearance calibration

There was no significant effect of the item’s serial position *F*(1,87) = 1.43, *p* = 0.234. Importantly, overall, a one-sample two-sided Bayesian *t*-test showed the average difference of appearance confidence from the proportion of errors was positively different than zero (M = 52.8, SD = 24.6, two-sided Wilcoxon test, *V* = 7627, *p* < 0.001, BF > 1e^+8^), suggesting that participants exhibited high degree of confidence that the probed item did not appear.

##### Location accuracy

Overall, location accuracy was poor (M = 40.2%, SD = 29.6), but above chance level (25%, one-sided Wilcoxon test, *V* = 4951, *p* < 0.001). There was no significant effect of serial position, *F*(1,87) < 1, *p* > 0.1.

#### Appearance Resolution

We calculated the Gamma correlation coefficient for appearance responses and appearance confidence, across all trials (i.e., the correct response was every trial that was reported as “appeared”). One participant’s data were excluded due to ceiling performance as none of the trials was reported as “not-appeared.” Overall, the resolution was high (M = 0.69, SD = 0.25) but should be taken cautiously due to the high proportion of correct responses and ceiling performance.

### Discussion

In this experiment, we asked participants whether they remember that the probed-item appeared. When they did remember the item, they were fairly calibrated (and slightly overconfident). In addition, both identity and location memory showed serial position effects, as the last item was the most accurate in all respects.

More importantly, we found that in about 11% of all trials, participants erroneously reported with relatively high confidence that the probe item did not appear. This number might not seem high; however, these appearance errors (or more likely rapid-forgetting errors) were found even though the conditions were optimal for remembering that the probed item appeared. Namely, memory load was low and within capacity, the items were presented in isolation at distinct locations for a relatively long duration, and there were no intrusions from previous trials. Thus, these results imply that observers can easily fail to remember a visually distinct and fully visible item even though VWM capacity is not exceeded. Furthermore, the relatively high confidence of subjects that the probed item did not appear, although admittedly falls below the confidence in “appeared” trials, demonstrates the underestimation of memory errors. That is, rather than being less confident that the item appeared, participants were more confident that the item did not appear.

These identity-appearance errors further point to the fragility of VWM. However, intriguingly, the location accuracy of those trials was above chance level, suggesting that participants had at least some degree of access to the item’s memory representation or that location memory was more accessible. This notion is consistent with the finding that in change-detection paradigms, observers are quite good at localizing the change but show difficulties in trying to report the identity of the changed item (Caplovitz et al., 2008; Hughes et al., 2012). Yet, before making strong conclusions in that regard, several alternative explanations should be considered. First, it may be possible that in some trials participants mistakenly reported that they did not remember the probed item, when in fact they did and were thus able to report its correct location. Second, participants’ strategy might account for some of these results. For example, participants might choose the location based on an “educated guess” by elimination if they remember more than one item and its location, or alternatively, they can choose an “empty” location—one that is not associated with any remembered item.

It is also possible that an old-new type question (i.e., item appeared or not) is more difficult and requires access to more information than the question of where the item appeared. There are more possibilities to choose from when trying to judge whether an item appeared (comparing the item against all possible memory traces) than making a decision regarding its location (one out of four possible locations, but see Makovski et al., 2010)^2^. Thus, participants might be prone to report “did not appear,” as it is harder to access the item’s identity, but its representation (and location information) is still accessible to some extent.

## Experiment 3

The results thus far showed that participants overestimate their VWM abilities in knowing where an item was presented (Experiment 1) and in knowing that an item was, in fact, presented (Experiment 2). In the final experiment, we wished to replicate these findings and extend them to another dimension of the task: the temporal domain.

**FIGURE 4:**
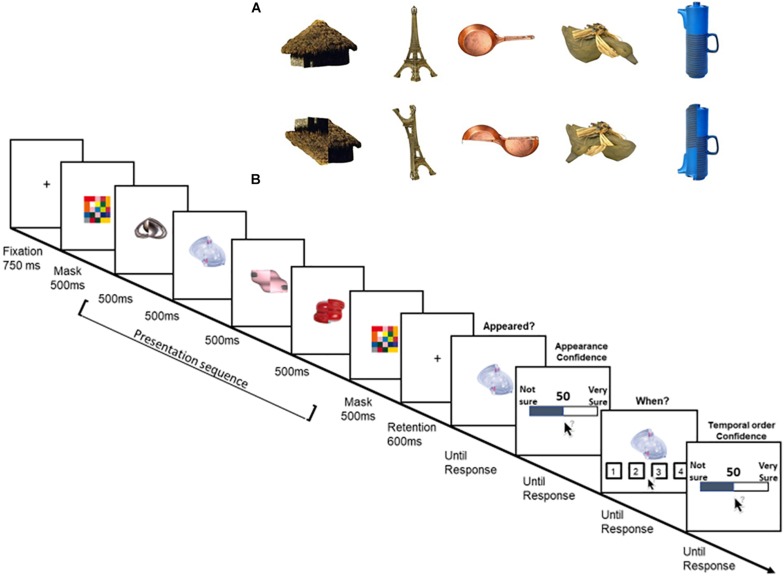
**(A)** Examples of items used in Experiment 3. Top row: high-semantic, intact items. Bottom row: low-semantic, distorted items. **(B)** Schematic illustration of a trial’s sequence in the low-semantic condition of Experiment 3.

Thus, the design of this experiment included subjective and objective questions about both the identity (appearance) and the temporal order of the probe. In order to prevent observers from using spatial locations as memory cues, each item was presented (separately) at the center of the screen. After the four items were presented, participants were now asked to report, first, whether they remembered the probed item and to rate their confidence on appearance, and second, what was the item’s serial position (i.e., was the probed item shown first, second, third, or fourth? Note that the item was always shown in the presentation sequence) and again to indicate their confidence in their temporal order response (Figure 4B). Same as in Experiment 2, the trial continued irrespective of the appearance response. Thus, we were able to also measure the temporal order subjective and objective measures in trials where participants did not remember the item’s identity.

The second goal of Experiment 3 was to test the role of semantics in the metacognitive processes of VWM. Indeed, a notable difference between complex, real-world objects and simple stimuli is that semantic meaning might be involved in VWM tasks, particularly when using real-world objects (Shoval et al., 2019). The role of meaning might be especially important in metacognition as reporting about explicit subjective judgments, while the probe is presented, could be biased by item’s label and meaning (e.g., “I haven’t seen this car”). Therefore, in order to directly test whether meaning plays a role in VWM and metacognition of VWM, two types of items were tested in Experiment 3: images of intact objects (high-semantic) and distorted versions of the same images (low-semantic).

Specifically, images of real-world objects were flipped 90° along their vertical or horizontal midline (Figure 4A). This simple manipulation kept most of the item’s visual properties but reduced its meaning. Indeed, there might not be fully meaningless object, and one might still “recognize” the identity of the distorted item (e.g., Figure 4A, the Eiffel tower) or attribute a meaning to what seems to be meaningless (e.g., the distorted pan, Figure 4A). However, in a previous study, several manipulation checks showed that participants were slower to verbally name these distorted objects, and these items were rated as less “meaningful” than their intact counterparts (Makovski, 2018). Furthermore, these low-semantic items were shown to considerably reduce VWM capacity (Shoval and Makovski, submitted) and were, therefore, good candidates to test the role of semantic in the metacognition of VWM.

### Method

The high-semantic objects were 600 images drawn from the same set of Experiment 1 (Figure 4A, top). The low-semantic objects were distorted versions of those images (Figure 4A, bottom). Specifically, half of the image was flipped along the vertical or horizontal midline. This allowed to disrupt the item’s meaning but to keep the visual statistics similar for both intact and distorted items (for further details and manipulation checks, see Makovski, 2018. The full stimuli set is publicly available at https://osf.io/3rn9k/).

Each trial began with the presentation of a black fixation cross against a white background for 750 ms. Then, a multicolored square was shown for 500 ms at the center of the screen followed by the memory items. The memory sequence included four items that were shown sequentially at the center of the screen, each for 500 ms. At the end of the sequence, the multicolored square was shown again for 500 ms before a 600 ms blank retention interval. Then, the probed item appeared at the center of the screen and participants were asked to indicate whether they remember that this item appeared, by pressing the keys “1” if they did or “2” if not. Note that same as in Experiment 2, the trial continued regardless the appeared or not response. Next, participants were asked to rate their confidence regarding that item’s appearance, in the same method as in Experiment 1. After this response was registered, the probed item re-appeared together with four black numbered frames (each 4.5° × 4.5°, located below fixation). The numbers (1,2,3,4 from left to right) were shown inside the frames and represented the serial position. Participants were asked to indicate the item’s serial position using the mouse. Then, they were asked to rate their confidence that they were correct in a similar way as before (Figure 4B).

Participants performed two 96-trials blocks (i.e., each serial position was tested 24 times). Each block consisted of either the high-semantic items or the low-semantic items. The starting order of the blocks was counterbalanced across participants. Before the task started, participants performed eight practice trials, four in each semantic condition. Every 32 trials, participants could take a short break. Twenty-nine new participants completed Experiment 2 (23 females, mean age 25.5).

### Results

#### Statistical Analyses

As before, for “appeared” trials, we used repeated-measures ANOVA with semantic level (high, low) and serial position (1–4) as a within-subject factor with Greenhouse–Geisser correction where needed. *Post hoc* comparisons were Bonferroni corrected. For the “not appeared” trials, we used LMM approach, similar to Experiment 2. The model for Experiment 3 was with two main effects: semantic (high, low) and serial position (1–4) and an interaction term as fixed effects, and participants as a random effect.

**FIGURE 5:**
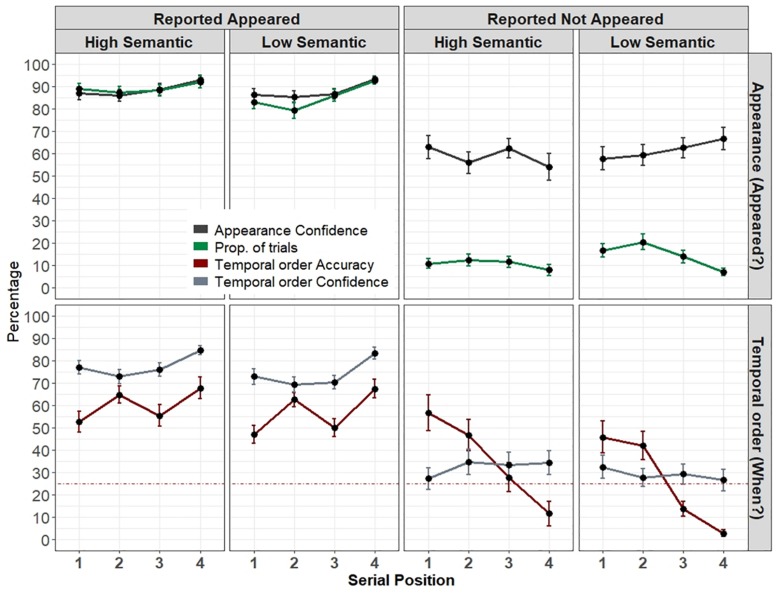
Experiment’s 3 results. Top: Proportion of trials and appearance confidence as a function of semantic level, reported appeared or not, and serial position. Bottom: Percentage of correct temporal order responses and mean confidence rating as a function of the item’s semantic level, reported appeared or not, and serial position. The dotted line represents the 25% chance level. Error bars represent one standard error of the mean.

Figure 5 depicts the results.

#### Proportion of Trials and Appearance Errors

Overall, the proportion of “appeared” trials was high (M = 87.2%, SD = 12.1). The complement, the proportion of appearance errors (12%) was similar to Experiment 2. Note that same as in Experiment 2, all probes were actually always presented on each trial. A repeated-measures ANOVA revealed a main effect of semantic-level *F*(1,28) = 6.7, *p* = 0.015, η_p_^2^ = 0.19, in that high-semantic items were more often reported as “appeared” (M = 89.1%, SD = 13.4) than low-semantic items (M = 85.3%, SD = 14.7). There was also a significant main effect of serial position, *F*(2.07,58.17) = 11.57, *p* < 0.001, η_p_^2^ = 0.29. *Post hoc* comparisons showed that the last position was the most accurate with the highest proportion of trials (all *p*’s < 0.007). These two factors interacted, *F*(3,84) = 5.04, *p* = 0.003, η_p_^2^ = 0.15. The interaction was mostly driven by a smaller proportion of “appeared” responses (and a larger proportion of appearance errors) for low semantic items that appeared in the second position (*p* = 0.008). Low-semantic items also produced a more pronounced serial effect, with fewer appearance errors for the last item than all other items (all *p*’s < 0.02) and fewer errors in the third item than the second (*p* = 0.035). These effects were not present in the high-semantic items as no other comparison reached significance.

#### Reported Appeared

##### Appearance confidence

The confidence that the item appeared was high (M = 88, SD = 12). The same repeated-measures ANOVA with semantic level (high, low) and serial position (1–4) as factors, showed only a serial position effect *F*(1.85,51.84) = 17.52, *p* < 0.001, η_p_^2^ = 0.38. *Post hoc* comparisons showed that only the last item’s appearance confidence was higher than all other positions (all *p*’s < 0.001).

##### Appearance calibration

Participants were well-calibrated (M = 1.07, SD = 9) and the calibration was not statistically different from zero [two-sided one-sample *t*-test, *t*(28) = 0.637, *p* = 0.529, BF = 0.238]. The analysis showed a main effect of semantic-level, *F*(1,28) = 4.69, *p* = 0.039, η_p_^2^ = 0.14, as participants were better calibrated for high-semantic items. There was also a significant interaction with serial position, *F*(3,84) = 4.07, *p* = 0.013, η_p_^2^ = 0.12, as participants were slightly overconfident in the first and second items of the low-semantic items (M = 3.2, M = 6), but were slightly underconfident in those items for high-semantic items (M = -2.1, M = -1.2). It should be noted again, however, that any conclusion about confidence bias in these results should be taken cautiously given the high-performance in the identity memory task.

##### Temporal order accuracy

Overall, the percentage of correct temporal order responses was moderate and above chance level [M = 58.4%, SD = 16.5, *t*(28) = 10.89, *p* < 0.001]. There was only a significant effect of serial position, *F*(3,84) = 10.22, *p* < 0.001, η_p_^2^ = 0.26. *Post hoc* comparisons showed that the last item was more accurate than any of the previous items (all *p*’s < 0.001) except for the second item. The second item was more accurate than the third and first items (*p*’s < 0.029). Further analysis suggested that this advantage of the second position possibly stems from a response bias (i.e., a large proportion of “second position” responses, M = 35%, SD = 9.2, likely because participants mostly guessed “2” whenever they were unsure).

##### Temporal order confidence

The overall temporal order confidence was relatively high (M = 75.7, SD = 14). The analysis showed a main effect of semantic-level, *F*(1,28) = 7.5, *p* = 0.011, η_p_^2^ = 0.21. The confidence was higher for high-semantic items (M = 77.5) than low-semantic items (M = 73.8). The analysis also showed a significant serial position effect, *F*(2.1,59.7) = 25.29, *p* < 0.001, η_p_^2^ = 0.14, in that the last item received the highest confidence (all *p*’s < 0.001).

##### Temporal order calibration

Similar to the spatial domain tested in Experiment 1, we found an overconfidence bias in the temporal domain as calibration was positively above zero [M = 17.3, SD = 12.5, *t*(28) = 7.4, *p* < 0.001, BF > 1e^+5^]. The analysis further showed a main effect of serial position, *F*(2.3,65.2) = 9.68, *p* < 0.001. η_p_^2^ = 0.25, resulting from the accurate calibration of the second position.

#### Reported Not Appeared

We now turn to examine the appearance confidence and calibration, as well as the temporal order accuracy, confidence, and calibration separately for the trials on which participants erroneously reported that the probed item did not appear.

##### Appearance confidence

Overall, the confidence that an item did not appear was relatively high (M = 66.2, SD = 26). There were no significant effects of neither semantic-level and serial position, and the two did not interact, all *F*’s < 1.3, all *p*’s > 0.1.

##### Appearance calibration

The overall difference between the confidence that the probed item did not appear, and the proportion of these errors was positively high and different than zero (M = 44.7, SD = 25.9, *V* = 400, *p* < 0.001, BF > 1e^+5^). That is, participants exhibited a high degree of confidence that the probed item did not appear. Again, there were no significant differences in both semantic and serial position, nor significant interaction, all *F*’s < 2.1, all *p*’s > 0.1

##### Temporal order accuracy

The overall temporal order accuracy was poor (M = 32.7%, SD = 37.35) and was not different than chance level (25%, Wilcoxon one-sided test, *V* = 7185.5, *p* = 0.054). The analysis showed a significant effect of serial position *F*(3,127) = 14.3, *p* < 0.001. *Post hoc* comparisons showed that the last item was worse than all other items (all *p*’s < 0.001) except for the third item. The third item was worse than the second and the first items (*p* < 0.006). No other comparison was significant.

##### Temporal order confidence

The confidence was overall low (M = 30.9, SD = 26.5). The analysis showed that none of the factors were significant, all *F*’s < 1, all *p*’s > 0.1.

##### Temporal order calibration

The overall calibration (M = -1.8, SD = 44) was not statistically different than zero (two-sided Wilcoxon test, *V* = 5318, *p* = 0.9, BF = 0.234). Yet, this does not suggest that participants were well-calibrated because there was also a robust serial position effect *F*(3,127) = 12.122, *p* < 0.001. *Post hoc* comparisons showed that last and third items (M = 24, M = 10) largely differed from the second and first items (M = -13, M = -20, all *p*’s < 0.016). Thus, the change from overconfidence to underconfidence (which evens out to zero) was driven only by the large decrease in the temporal order accuracy across serial positions (probably because of the tendency to guess 1 or 2 when not knowing) that was not companied by a change in confidence (see Figure 5, bottom right).

##### Appearance resolution

We calculated the Gamma correlation coefficient for appearance responses and appearance confidence separately for each semantic condition. Four participants’ data were excluded due to ceiling performance, as only one or no trials were reported as “not-appeared.” Overall and similar to Experiment 2, the resolution was high (M = 0.69, SD = 0.27) and should be taken cautiously due to ceiling performance. There was also no difference between high-semantic (M = 0.70, SD = 0.28) and low-semantic items [M = 0.68, SD = 0.28, paired two-sided *t*-test, *t*(24) = 0.88, *p* = 0.384].

##### Temporal order resolution

We also calculated the Gamma correlation coefficient for temporal order responses and its confidence across all trials, separately for each semantic condition. Overall, the temporal order resolution was high and similar to Experiment 1 (M = 0.48, SD = 0.22). There was also a significant difference between high-semantic items (M = 0.51, SD = 0.22) and low-semantic items [M = 0.45, SD = 0.23, paired two-sided *t*-test, *t*(28) = 2.36, *p* = 0.025].

### Discussion

As detailed below, the results of Experiment 3 provided further generalization to our previous experiments, and extend the relevant findings to the temporal domain. From a general metacognitive view, the results of Experiment 3 were quite similar to those of Experiments 1 and 2. The temporal order task produced an overconfidence bias. The discrimination (of confidence judgments) between correct and incorrect temporal order responses (i.e., resolution) was high and better particularly for the high-semantic items, and both item types exhibited high resolution regarding the item’s appearance. On the other hand, in this experiment, the appearance calibration was influenced by both the item type and its serial position.

The current findings also replicated the same appearance errors observed in Experiment 2, as participants erred in about 12% of the trials, even though the memory set-size was within VWM capacity limits and there was no proactive interference. Importantly, low-semantic items were more susceptible to these errors compared to the high-semantic items, especially when these items appeared at the beginning of the memory array. Similar to Experiment 2, these errors were followed with high confidence that the probed item did not appear. This critically points to a gap in subjective judgments’ reliability (Bona et al., 2013; Adam and Vogel, 2017), suggesting that participants were “confidently-blind” to their errors regarding whether the probed item appeared or not. In contrast to Experiment 2’s results, however, when the item was reported as “did not appear”—the overall temporal order accuracy was not better than chance. Nevertheless, the overall accuracy was lower in Experiment 3 than in Experiment 2 [M = 47.7 vs. 52.9, one-sided independent-samples *t*-test, *t*(63) = 2.1, *p* = 0.019] and thus it is possible that this overall reduction in performance can account for the difference between the two experiments.

More importantly, we found that meaning played a significant role in VWM and in the metacognitive processes of VWM. Consistent with previous findings, meaning enhanced VWM performance (Brady et al., 2016; Shoval and Makovski, submitted). There were fewer appearance errors for high-semantic than for low-semantic items. Yet, participants not only better remembered these items, but they were also more confident, exhibited better resolution in the temporal order task, and were better calibrated for the item’s appearance when asked about high-semantic items compared to low-semantic items.

## General Discussion

The present study explored the metacognitive processes in VWM. Unlike other VWM studies, which typically tested simple stimuli (colors, orientation, etc.), the present study used unique (non-repeating) images of real-world objects as ecological stimuli. These stimuli enabled us to minimize the influence of proactive interference and to test subjective judgments with minimum interference from previous trials intrusions. Experiment 1 examined location accuracy and metacognitive measures for a six-item memory array. Experiment 2 used a four-item memory array for a spatial memory task and directly examined the confidence of observers in their memory of item’s appearance. Experiment 3 investigated the subjective and objective performance for both the item’s identity and temporal order, as well as the role of the item’s semantics in memory and metacognitive performance.

From a general metacognitive perspective, we replicated common findings from other cognitive tasks. Namely, participants consistently exhibited an overconfidence bias, along with moderate resolution (Koriat, 2007; Fiedler et al., 2019). Overconfidence seems to be a persistent and typical finding in VWM and other domains (Pallier et al., 2002; Dentakos et al., 2019). As abovementioned, the resolution estimates for meaningful objects were higher than the resolution reported in previous studies (but see, Masson and Rotello, 2009). Taken together, calibration and resolution may dissociate, with adequate resolution and poor calibration (i.e., overconfidence) as they are the product of different mechanisms (Koriat, 2007).

Moreover, the findings from Experiments 2 and 3, and specifically, the highly confident memory failures—in which participants erroneously reported that the probed item did not appear, further challenge the reliability of subjective judgments. Previous studies showed mixed results in that regard, where in one study observers rarely exhibited memory failures with high confidence (Rademaker et al., 2012), whereas in another study, observers were mostly blind to their memory failures (Adam and Vogel, 2017). The current results are in line with the latter and challenge the reliability of assessments regarding one’s own working memory performance—it resulted in a consistent overestimation of performance along with underestimation of memory failures, even within capacity limits and in the absence of intrusions from previous trials. That is, the assessment of VWM content seems to be subjected to biases, such as “blind” errors, and the overestimation of location and temporal memory performance.

It is also noteworthy that while objective accuracy was better for the last appearing items in the spatial, temporal, and identity tasks, participants were well-calibrated for these memory items only in the identity and spatial tasks. However, the robustness of this finding is not very clear because for the spatial task it was driven by only two data points under a high memory-load condition and calibration in the identity tasks is difficult to assess because of the overall high performance in this task. Thus, additional research is still needed in order to establish the cases in which participants are well-calibrated.

Similarly, future research should scrutinize the phenomenon in which observers report that fully visible items did not appear even though VWM was not full (e.g., Chen et al., 2019). This seems to be an intriguing and unexpected effect as one would expect that four real-world, visually distinct objects should be easily remembered. Furthermore, more research is specifically required for clarifying the finding that observers had better than chance knowledge about items that they reported as did-not-appear in the spatial memory task of Experiment 2, but not in the temporal memory task of Experiment 3.

Only a few studies have previously examined how the task requirements (e.g., spatial, temporal, or identity tasks) affect metacognitive measures, as most studies usually rely on the probed-item identity as the objective measure (e.g., “is this color old or new?”). A notable exception is a study employing a metacognitive framework that focused on age differences in a spatial working memory task and found that spatial information resulted in better performance compared to identity information. Furthermore, while identity accuracy decreased as a function of the memory set-size, the location accuracy remained mostly unaffected. However, from a metacognitive perspective, the resolution of identity information was higher than the resolution of location information (Exp. 1a, 1b, Thomas et al., 2012). Given the methodological differences between this and the current study, it is hard to draw any conclusions. But it also important to note that improved performance may result in higher resolution (Rouault et al., 2018), and eventually identity information is often associated with a specific location (e.g., Toh et al., 2020).

Other studies that examined the metacognitive monitoring in temporal tasks showed mixed results. One study found that observers were accurate in reproducing temporal information and were aware of their errors (Akdogan and Balcı, 2017). Consistent with the current results, another study found that participants were largely unaware of their errors and without explicit feedback on errors, participants overestimated their performance in a temporal task (Riemer et al., 2019). Taken together, the current results seem to suggest that the basic metacognitive principles and biases apply regardless of the exact task at hand.

In addition, the findings from Experiment 3 imply that stimuli meaning and semantics play a role in VWM and metacognitive judgments. The results showed that high-semantic items were more accurate and less prone to appearance errors. They were also rated with greater confidence than distorted, low-semantic items. The resolution for high-semantic items in a temporal task was better than for low-semantic items, but both item types exhibited the same degree of false-confidence when participants reported that the probed-item did not appear.

This pattern suggests that observers were able to add a semantic label to an image—one which improved the following recognition of that image (see Brady et al., 2016; Shoval and Makovski, submitted). Meaning could improve VWM performance by different routes. It is possible, for instance, that meaning acts as a conceptual hook: the label of an item essentially adds another level of available information, which can later be used as a “retrieval” cue (e.g., Konkle et al., 2010). Another possibility is that relying on previous knowledge (i.e., item’s semantics) might reduce the amount and complexity of the visual information needed to actively maintain the item’s representation in VWM (because previous knowledge about an item is probably associated with at least some “prototypical” visual features and therefore reduces the information entropy).

This involvement of semantics in VWM could not only ease the maintenance which in turn, allows for better recognition, but could also make the item’s visual representation and associated information more accessible for later judgments (see Berry et al., 2013, for findings that suggest that meaningful memory items improve metacognition). Be that as it may, these findings suggest that the use of real-world objects in VWM tasks lead to the involvement of long-term memory in that task, and thus should be taken into account when trying to isolate VWM capacity measures (Shoval and Makovski, submitted).

In sum, the metacognition of VWM of real-world objects seems to follow a similar pattern of metacognitive results, especially those suggesting overestimation of performance as well as underestimation of “blind” errors. These biases seem not to be task-specific as they were found in temporal, spatial, and identity VWM tasks. We further found that the use of meaningful, complex items, which improved VWM performance, also increased the confidence ratings as well as the metacognitive resolution in a temporal order task. Together these findings challenge the consistency of subjective judgments in VWM and calls for caution in the use of meaningful objects in VWM tasks when attempting to isolate VWM performance.

## Data Availability Statement

The data and tasks supporting this article are available on Open Science Framework (https://osf.io/nfkj9/).

## Ethics Statement

The studies involving human participants were reviewed and approved by the Department of Psychology and Education Ethics Committee, The Open University of Israel. The patients/participants provided their written informed consent to participate in this study.

## Author Contributions

TM, YS, and TS contributed to the conception and design of the study. YS and TS performed the statistical analysis. TS wrote the first draft of the manuscript. YS and TM provided critical comments on the manuscript. All authors contributed to the manuscript revision, and read and approved the submitted version.

## Conflict of Interest

The authors declare that the research was conducted in the absence of any commercial or financial relationships that could be construed as a potential conflict of interest.

## Footnotes

1 https://bradylab.ucsd.edu/stimuli.html

2 We thank Dr. Dominique Lamy for raising this possibility.
